# The carbonic anhydrase IX inhibitor SLC-0111 sensitises cancer cells to conventional chemotherapy

**DOI:** 10.1080/14756366.2018.1532419

**Published:** 2018-10-26

**Authors:** Elena Andreucci, Jessica Ruzzolini, Silvia Peppicelli, Francesca Bianchini, Anna Laurenzana, Fabrizio Carta, Claudiu T. Supuran, Lido Calorini

**Affiliations:** a Department of Clinical and Experimental Biomedical Sciences “Mario Serio”, Section of Experimental Pathology and Oncology, University of Florence, Florence, Italy;; b Department of NEUROFARBA, University of Florence, Florence, Italy;; c Center of Excellence for Research, Transfer and High Education DenoTHE, University of Florence, Florence, Italy

**Keywords:** Chemotherapy, drug resistance, SLC-0111, CAIX inhibitor, combined therapy

## Abstract

Drug combination represents one of the most accredited strategies of cancer therapy able to improve drug efficacy and possibly overcome drug resistance. Among the agents used to complement conventional chemotherapy, carbonic anhydrase IX (CAIX) inhibitors appear as one of the most suitable, as markers of hypoxic and acidic cancer cells which do not respond to chemo- and radiotherapy. We performed preclinical in vitro assays to evaluate whether the SLC-0111 CAIX inhibitor co-operates and potentiates the cytotoxic effects of conventional chemotherapeutic drugs in A375-M6 melanoma cells, MCF7 breast cancer cells, and HCT116 colorectal cancer cells. Here, we demonstrate that the SLC-0111 CAIX inhibitor potentiates cytotoxicity of Dacarbazine and Temozolomide currently used for advanced melanoma treatment. SLC-0111 also increases breast cancer cell response to Doxorubicin and enhances 5-Fluorouracil cytostatic activity on colon cancer cells. These findings disclose the possibility to extend the use of CAIX inhibitors in the combination therapy of various cancer histotypes.

## Introduction

Therapy resistance represents the main issue for cancer treatment and obstacles the good outcome of cancer patients. Cancer cells develop resistance to almost all chemotherapeutic agents via different mechanisms, for instance reducing drug accumulation and increasing drug export, altering drug targets and signalling transduction molecules, increasing repair of drug-induced DNA damage, and promoting apoptosis evasion programs^1^. Drug resistance consists of a lack of response to a specific drug, and it may depend on special resistant subpopulation of cancer cells that cause a poor initial treatment response without prior exposure to anticancer agents—“intrinsic resistance”—or is acquired as a cellular adaptation, with an initial good treatment response followed by poor results and a devastating outcome—“acquired resistance”^2^. The issue of drug resistance also regards the so-called personalised medicine, developed from the genetic information collected from tumour tissues, based on targeted anticancer drugs that often involves kinase inhibitors^2^. Thus, despite the significant progresses in the development of anticancer therapeutic strategies, involving either conventional or targeted therapies, drug resistance still represents a common phenomenon in tumour-bearing patients.

The development of drug resistance leads to consider the need for drug combination strategy. Complementary therapy may reduce the incidence of resistance as increasing drug efficacy and the overall survival rate of treated patients. This is why a large part of the effort dedicated to cancer therapy is directed towards the study for drug combinations.

Tumour microenvironment has emerged as a key player in the development of chemoresistance and in malignant progression^3^^,^^4^. For most tumours, it is characterised by hypoxia and acidosis, both conditions that profoundly influence cancer cell biology and inhibit therapy response^5–7^. Identifying the agents of microenvironment-mediated progression and drug resistance might yield information to avoid them. Among them, carbonic anhydrase (CA, EC 4.2.2.1) IX has increasingly drawn the attention of cancer researchers. CAIX, a tumour-associated metalloenzyme that catalyzes the reversible formation of HCO_3_^−^ and H^+^ ions from H_2_O and CO_2_, basically maintains a favourable intracellular pH for tumour cell survival and growth and is correlated with cancer cell migration, invasion, and maintenance of stemness properties^8^. CAIX expression is promoted by hypoxia-inducible factors 1α (HIF-1α) in the hypoxic regions within the tumour mass^9^ and also by extracellular acidic microenvironment via HIF-1α-independent mechanisms^10^^,^^11^. We have previously demonstrated the increased CAIX expression in melanoma, breast, and colorectal cancer cells transiently and chronically exposed to an extracellular acidic microenvironment (pH 6.7 ± 0.1). Extracellular acidosis represents a “diabolic” characteristic of most solid tumours that correlates with aggressive phenotypes and therapy resistance. Moreover, we also demonstrated that the CAIX inhibitor SLC-0111 is able not only to prevent such CAIX increased expression but also to selectively induce the apoptotic program in A375-M6 melanoma cells, MCF7 breast cancer cells, and HCT116 colorectal cancer cells transiently and chronically exposed to extracellular acidosis, without showing any cytotoxic effect in the population maintained under standard pH condition (pH 7.4 ± 0.1)^10^. Thus, CAIX expression represents a common cancer cell adaptation to changes in tumour microenvironment, such as hypoxia and acidosis, both involved in cancer progression and resistance. CAIX expression in human tumour samples is always associated with tumour progression and poor prognosis^12–16^ and its block through chemical inhibitors, either as a single treatment or in combination with radiotherapy, significantly reduces tumour growth in vivo^17^^,^^18^. Moreover, CAIX targeting by Acetazolamide treatment enhances the anti-angiogenic effect of Bevacizumab^19^.

In this study, we have investigated if CAIX targeting may complement conventional chemotherapy in the treatment of melanoma, breast, and colon cancer. We demonstrated that SLC-0111, a novel CAIX inhibitor, is able to synergise with Dacarbazine and its derivative Temozolomide, Doxorubicin and 5-Fluorouralcil in the treatment of melanoma, breast, and colorectal cancer, respectively, which, as reported in our previous paper^10^, express a significant level of mRNA and protein of CAIX also in normoxia.

## Materials and methods

### Cell cultures

Human melanoma cell line A375-M6, breast carcinoma MCF7 cell line, and colorectal carcinoma HCT116 cell line were maintained in DMEM 4,5 g/L glucose and 2 mM L-glutamine supplemented with 10% fetal bovine serum (Euroclone, Milan, Italy) as previously described^10^. CAIX inhibitor SLC-0111, developed in the laboratory of Professor Claudiu T. Supuran (NEUROFARBA Department, University of Florence, Italy) and previously described^10^, was used at 100 µM dose alone or in combination with Dacarbazine (Sigma Aldrich, Saint Louis, Missouri, USA) and Temozolomide (MedChemExpress, Sollentuna, Sweden) for melanoma cells, Doxorbicin (MedChemExpress) for breast cancer cells, or 5-Fluorouracil (MedChemExpress) for colorectal cancer cells, to evaluate a potential enhanced response of tumor cells to conventional chemotherapeutics. Dacarbazine, Temozolomide, Doxorubicin, and 5-Fluorouralcil were used at a lower dose than the half maximal inhibitory concentration (IC_50_) (data not shown).

### Cell death evaluation

Cell death was determined by flow cytometer analysis using Annexin V FITC/APC or FITC-conjugated (Immunotools GmbH, Germany) and PI (Sigma-Aldrich) according to the manufacturer's protocol. Briefly, cells were harvested with Accutase (Eurolone), collected in flow cytometer tubes (1 × 10^5^ cells/tube), washed in PBS and incubated 15 min at 4 °C in the dark with 100 µl Annexin Binding buffer (100 mM HEPES, 140 mM NaCl, 25 mM CaCl2, pH 7.4), 1 µl of 100 µg/ml PI working solution, and 5 µl Annexin V FITC/PI-conjugated. Each sample was added with Annexin Binding Buffer to reach 500 µl volume/tube. Samples were then analyzed at BD FACSCanto (BD Biosciences, Franklin Lakes, New Jersey, USA). Cellular distribution depending on Annexin V and/or PI positivity allowed the measure of the percentage of viable cells (Annexin V and PI negative cells), early apoptosis (Annexin V positive and PI negative cells), late apoptosis (Annexin V and PI positive cells), and necrosis (Annexin V negative and PI positive cells). Alternatively, cell death was evaluated using Trypan blue exclusion test counting cells with Burker’s chamber at an optical microscope.

### Western blot analysis

Cells were lysed in RIPA buffer (Merck Millipore, Vimodrone, MI, Italy) containing PMSF (Sigma-Aldrich, Saint Louis, Missouri, USA), sodium orthovanadate (Sigma-Aldrich), and protease inhibitor cocktail (Calbiochem, San Diego, CA, USA), sonicated and centrifuged 15 min at 14,000 rpm at 4 °C. Equal amounts of protein were separated on Bolt^®^ Bis-Tris Plus gels, 4–12% precast polyacrylamide gels (Life Technologies, Monza, Italy). Fractionated proteins were transferred to a PVDF membrane using the iBlot 2 System (Life Technologies). Following 1-h blocking with Odyssey blocking buffer (Dasit Science, Cornaredo, MI, Italy), membrane was probed overnight at 4 °C with anti-cleaved caspase 3 antibody (Origene, Rockville, MD, USA), and 1 h at room temperature with goat anti-rabbit IgG Alexa Flour 750 antibody (Invitrogen, Monza, Italy). Membrane was visualised using the Odyssey Infrared Imaging System (LI-COR^®^ Bioscience, Lincoln, Nebraska USA). Anti-tubulin antibody (Sigma-Aldrich) was used to assess equal amount of protein loaded in each lane.

### Colony formation assay

2 × 10^2^ cells were seeded in six-well plate and treated 14 days with 100 µM SLC-0111 alone or in combination with 170 µM Temozolomide for A375-M6 melanoma cells, 10 nM Doxorubicin for MCF7 breast cancer cells, or 1 µM 5-Fluorouracil for HCT116 colorectal cancer cells. Developed colonies were counted upon 20 min-fixation in 4% paraformaldehyde at 4 °C and 30 min-staining with Crystal violet solution at room temperature. Colony diameter mean was calculated using ImageJ software.

### Statistical analysis

The experiments were performed at least three times for a reliable application of statistics. Statistical analysis was performed with GraphPad Prism software. Values are presented as mean ± SD. N value represents the number of biological replicates. One- and Two-way ANOVA were used to evaluate the statistical significance.

## Results

### SLC-0111 CAIX inhibitor sensitises melanoma cells to Dacarbazine and Temozolomide treatment

**Figure 1.**
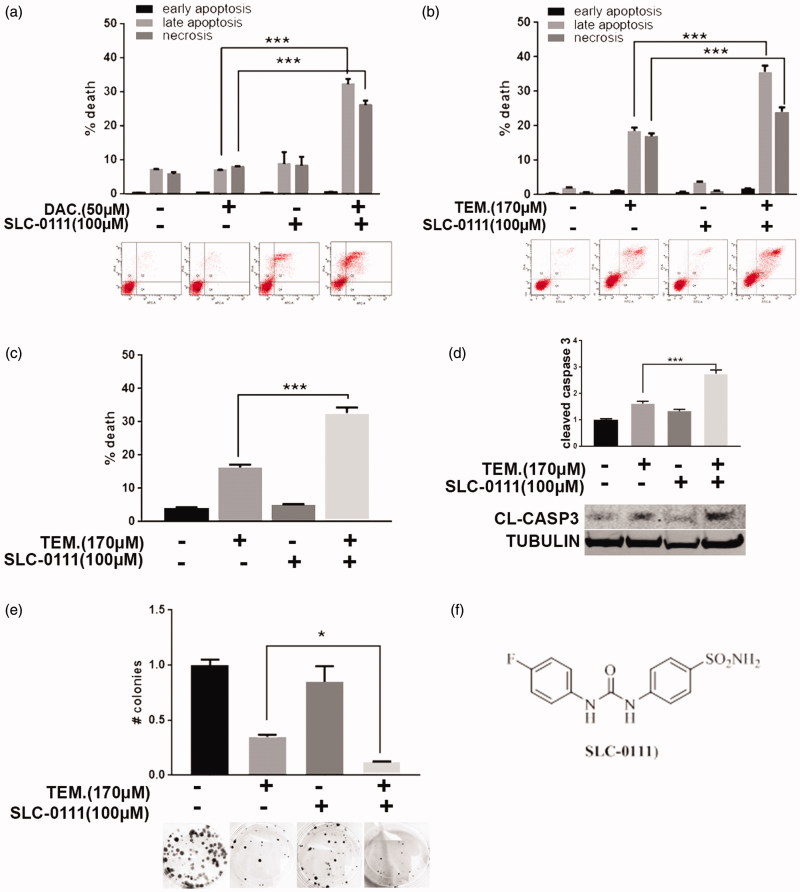
SLC-0111 sensitises melanoma cells to Dacarbazine and Temozolomide treatment. (a) Representative plots of Annexin V/PI assay of A375-M6 treated for 96 h with either 50 µM Dacarbazine, or 100 µM SLC-0111, or the combination of the two (lower) and relative quantification chart of early apoptosis, late apoptosis and necrosis ratio (upper). ***p < 0.001 Two-way ANOVA, *N* = 3. (b) Representative plots of Annexin V/PI assay of A375-M6 treated for 72 h with either 170 µM Temozolomide, or 100 µM SLC-0111, or the combination of the two (lower), and relative quantification chart of early apoptosis, late apoptosis, and necrosis ratio (upper). ***p < 0.001 Two-way ANOVA, *N* = 3. (c) Quantification chart of Trypan Blue exclusion assay of A375-M6 treated for 72 h with either 170 µM Temozolomide, or 100 µM SLC-0111, or the combination of the two. ***p < 0.001 One-way ANOVA, *N* = 3. (d) Quantification chart (upper) and representative image (lower) of western blot analysis of cleaved caspase 3 of A375-M6 treated 72 h with 170 µM Temozolomide and 100 µM SLC-0111 alone or combined. Tubulin used as loading control. ***p < 0.001, Two-way ANOVA, *N* = 3. (e) Quantification chart (upper) and representative pictures (lower) of colony formation assay of A375-M6 treated for 14 days with 170 µM Temozolomide and 100 µM SLC-0111 alone or in combination. *p < 0.05, One-way ANOVA, *N* = 3. (f) Chemical structure of SLC-0111.

As previously described, the CAIX inhibitor SLC-0111 does not affect cancer cell viability under standard pH condition^10^. To evaluate a possible effect of this compound in sensitizing cancer cells to conventional chemotherapy, we combined SLC-0111 with chemotherapeutics agents currently used in the clinic. In particular, we treated A375-M6 melanoma cells with sub-lethal dose of Dacarbazine alone or in combination with SLC-0111 to evaluate cell viability with different in vitro assays. As shown by Annexin V/PI assay (Figure 1(a)), A375-M6 treated for 96 h with 50 µM Dacarbazine or 100 µM SLC-0111 alone are not significantly affected in terms of viability, whereas the combined therapy induces significant increase of late apoptosis phase and necrosis. The 72 h-treatment with 170 µM (<IC50 dose) of the Dacarbazine derivative Temozolomide gives similar results because the combination of this alkylating agent with the SLC-0111 CAIX inhibitor significantly augmented cell death percentage, in particular, late apoptosis phase and necrosis (Figure 1(b)). These data are confirmed with Trypan Blue exclusion test (Figure 1(c)) and cleaved caspase 3 levels detected by western blot analysis (Figure 1(d)), both showing that the percentage of dead cells upon Temozolomide treatment is almost doubled when combined with SLC-0111. To further prove such data, we extended Temozolomide and SLC-0111 treatment to 14 days by performing a colony formation assay (Figure 1(e)) and observed similar results: in particular, while the number of developed colonies upon SLC-0111 treatment is comparable to control, the decrease obtained with Temozolomide treatment alone is further enhanced when used in combination with the CAIX inhibitor.

### SLC-0111 increases cytotoxic effect of Doxorubicin in breast cancer cells

**Figure 2.**
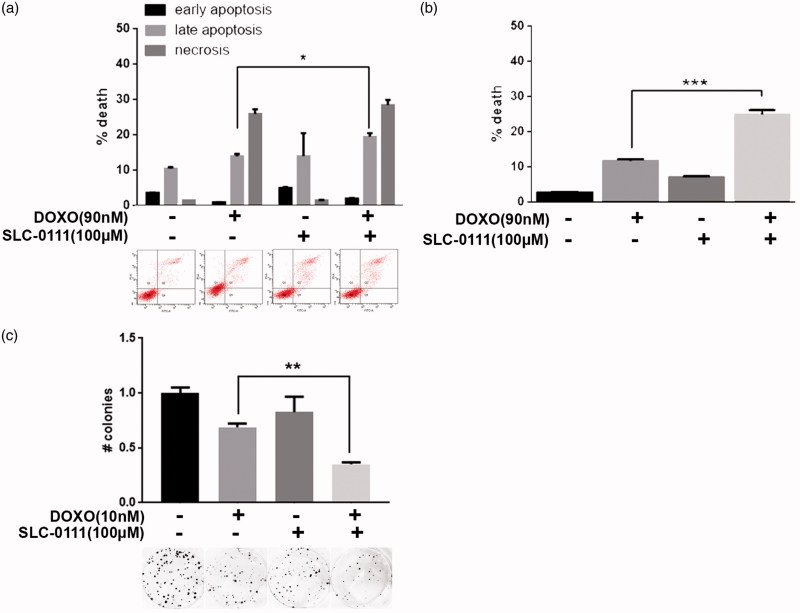
SLC-0111 increases cytotoxic effect of Doxorubicin in breast cancer cells. (a) Representative plots (lower) and relative quantification chart (upper) of Annexin V/PI assay of MCF7 treated for 48 h with either 90 nM Doxorubicin, or 100 µM SLC-0111, or the combination of the two. *p < 0.05, Two-way ANOVA, *N* = 3. (b) Quantification chart of Trypan Blue exclusion assay of MCF7 treated for 48 h with either 90 nM Doxorubicin, or 100 µM SLC-0111, or the combination of the two. ***p < 0.001 One-way ANOVA, *N* = 3. (c) Representative pictures (lower) and relative quantification chart (upper) of colony formation assay of MCF7 breast cancer cells treated for 14 days with either 10 nM Doxorubicin, or 100 µM SLC-0111, or the combination of the two. **p < 0.01, One-way ANOVA, *N* = 3.

To verify if the chemotherapy-sensitizing effect shown by the CAIX inhibitor in melanoma cells is extendable even in other tumour histotypes, we treated MCF7 breast cancer cells with 90 nM (<IC50 dose) of Doxorubicin alone or in combination with 100 µM SLC-0111 for 48 h and observed that the combined therapy significantly increases cell death percentage. We observed using Annexin V/PI assay that the addition of SLC-0111 to Doxorubicin treatment induces a slight but significant increase of late apoptosis phase (Figure 2(a)), result confirmed using Trypan Blue exclusion test (Figure 2(b)). Extending the treatment to 14 days in colony assay, we obtained similar data since while the SLC-0111 treatment alone does not significantly vary the colony number, the decrease obtained with Doxorubicin is further enhanced when used in combination with the CAIX inhibitor (Figure 2(c)).

### SLC-0111 increases 5-Fluorouracil response in colorectal cancer by reducing cell proliferation

**Figure 3.**
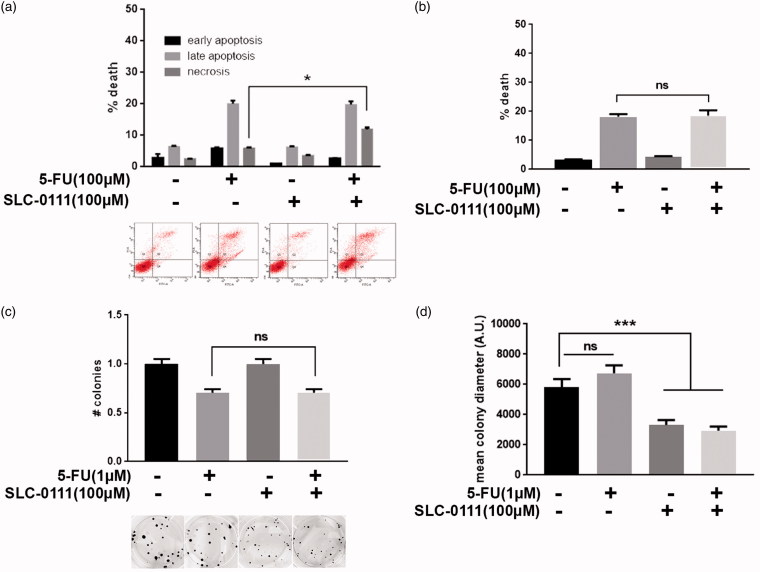
SLC-0111 increases 5-Fluorouracil response in colorectal cancer by reducing cell proliferation. (a) Representative plots (lower) and relative quantification chart (upper) of Annexin V/PI assay of HCT116 treated for 24 h with either 100 µM 5-Fluorouracil, or 100 µM SLC-0111, or the combination of the two. *p < 0.05, Two-way ANOVA, *N* = 3. (b) Quantification chart of Trypan Blue exclusion assay of HCT116 treated for 24 h with either 100 µM 5-Fluorouracil, or 100 µM SLC-0111, or the combination of the two. One-way ANOVA, *N* = 3. (c) Representative pictures (lower) and relative quantification chart (upper) of colony formation assay of HCT116 colorectal cancer cells treated for 14 days with either 1 µM 5-Fluorouracil, or 100 µM SLC-0111, or the combination of the two. One-way ANOVA, *N* = 3. (d) Quantification chart of the diameter mean of HCT116 colonies grown and treated for 14 days with either 1 µM 5-Fluorouracil, or 100 µM SLC-0111, or the combination of the two. ***p < 0.001, One-way ANOVA, *N* = 3.

HCT116 colorectal cancer cells were treated with 100 µM 5-Fluorouracil (<IC50 dose) alone or in combination with 100 µM SLC-0111 for 24 h to evaluate if the CAIX inhibitor increases cancer cell sensitivity to this chemotherapeutic agent. In contrast with what observed in melanoma and breast cancer cells, the combined treatment in colorectal cancer seems to have less efficacy in sensitizing HCT116 cells to conventional chemotherapy, at least in terms of cell viability. Indeed, by Annexin V/PI assay (Figure 3(a)), we observed a slight but significant increase in necrotic cell fraction when 5-Fluorouracil is combined with SLC-0111, compared to the single treatment, but by assessing cell death with Trypan blue exclusion test, we were not able to obtain comparable results since no significant variation in terms of cell viability occurs between combined and single treatment (Figure 3(b)). Finally, by using colony assay, we observed that, in accordance with Annexin V/PI and Trypan Blue results, the colony number does not significantly decrease in combined treatment compared to 5-Fluorouracil alone (Figure 3(c)) while the co-treatment with SLC-0111 significantly affects cancer cell proliferation as shown by the decreased diameter of the colonies upon CAIX inhibitor treatment (Figure 3(d)). Thus, SLC-0111 increases 5-Fluorouracil response in HCT116 by slowing down cancer cell proliferation without altering its cytotoxic potential.

## Discussion

We have previously demonstrated the increased CAIX expression in melanoma, breast, and colorectal cancer cells transiently and chronically exposed to an extracellular acidic microenvironment (pH 6.7 ± 0.1). Extracellular acidosis represents a “diabolic” characteristic of most solid tumours that correlates with aggressive phenotypes and therapy resistance^20–22^. Moreover, we also demonstrated that SLC-0111 is able not only to prevent such CAIX increased expression but also to selectively induce the apoptotic program in A375-M6 melanoma cells, MCF7 breast cancer cells, and HCT116 colorectal cancer cells transiently and chronically exposed to extracellular acidosis, without showing any cytotoxic effect in the population maintained under standard pH condition (pH 7.4 ± 0.1)^10^.

Herein, we have verified whether the CAIX inhibitor SLC-0111 we used may also affect the viability of cancer cells grown in normoxia and standard pH, acting as anticancer potentiating agent of standard chemotherapy. Metastatic melanoma, including the BRAF-mutated subgroup, is very resistant to both traditional chemotherapy and targeted therapy, leaving most of the patients undergoing resistance without any other therapeutic strategies. To date, Dacarbazine, a guanine methylating agent at the O-6 and N-7 positions, remains the reference standard treatment for stage IV melanoma. Still, it has been reported that Dacarbazine-treated patients often undergo resistance^23–25^. Here, we demonstrate that SLC-0111 CAIX inhibitor sensitises A375-M6 melanoma cells to Dacarbazine treatment, revealing a synergistic effect compared to the single treatments (Figure 1(a)).

More recently, Temozolomide, a Dacarbazine derivative, has been introduced as an oral alternative for patients with advanced metastatic melanoma^23^^,^^26^. Here, we demonstrate that even Temozolomide cytotoxicity is potentiated by the SLC-0111, thus melanoma cell death fraction was significantly increased compared to Temozolomide treatment alone (Figure 1(b–e)). Hence, this CAIX inhibitor significantly improves cell death of melanoma cells exposed to Dacarbazine or Temozolomide.

To further extend our observation to breast cancer, we used MCF7 cell line. For breast cancer patients, Doxorubicin treatment is considered the most effective chemotherapeutic agent although resistance development is common and represents a major obstacle to successful patient outcome^27–30^. We demonstrate that even in this case the addition of the SLC-0111 CAIX inhibitor to Doxorubicin treatment enhance its cytotoxic effect by increasing cell death fraction compared to single treatment (Figure 2(a–c)).

To investigate the potential effects of SLC-0111 on colorectal cancer, we used HCT116 cells treated with 5-Fluorouracil (5-FU), a pyrimidine analogue working as antimetabolites, being able to block the thymidylate synthase activity and consequently the DNA synthesis. 5-FU is currently used in the clinic for colorectal cancer representing the major treatment for advanced disease, but therapy resistance occurrence remains the major issue for cancer patients^31^^,^^32^. Here, we used the SLC-0111 inhibitor to evaluate if even in this tumour histotype the CAIX targeting may improve conventional anticancer treatment response. We observed that SLC-0111 significantly reduces tumour cell proliferation (Figure 3(c–d)), let us hypothesise it may contribute to increase 5-FU response working not on cytotoxicity—like in melanoma and breast cancer—but rather adding a cytostatic effect to 5-FU treatment alone.

This in vitro preclinical investigations show the ability of the CAIX inhibitor SLC-0111—at micromolar concentration and under normoxia condition—to potentiate anticancer effects of chemotherapy in melanoma, breast, and colon cancer cells. We speculate that this chemotherapy potentiation can be due to the intracellular pH increase occurring in cancer cells treated with SLC-0111, as previously reported^10^. Indeed, pH variation in both extracellular and intracellular compartments critically influences the destiny of chemotherapeutic agents. For instance, weak base drugs such as Doxorubicin reduce their cell permeability and efficacy in the presence of an acidic microenvironment, whereas weak acids like 5-Fluorouracil tend to concentrate in more alkaline environments such as intracellular compartments^33^. The efficacy showed by SLC-0111 on the various tumour histotypes used in this study suggests a possible common anticancer mechanism that might be used for an easy translation in vivo. In fact, this compound completed Phase I clinical trials and is presently in Phase II trials^34^.
